# Patient safety and quality of care in mental health: a world of its own?

**DOI:** 10.1192/pb.bp.116.055327

**Published:** 2017-10

**Authors:** Danielle D'Lima, Mike J. Crawford, Ara Darzi, Stephanie Archer

**Affiliations:** 1 University College London; 2 Imperial College London

## Abstract

Quality and safety in healthcare, as an academic discipline, has made significant progress over recent decades, and there is now an active and established community of researchers and practitioners. However, work has predominantly focused on physical health, despite broader controversy regarding the attention paid to, and significance attributed to, mental health. Work from both communities is required in order to ensure that quality and safety is actively embedded within mental health research and practice and that the academic discipline of quality and safety accurately represents the scientific knowledge that has been accumulated within the mental health community.

With repeated examples of failure across the healthcare system,^1,2^ there has long been a need to understand how we can better uphold and improve on the quality and safety of care that is being provided to our patients. There is a gap between the policy and guidelines generated from research evidence and the practice of medical, nursing and allied health professionals. This gap is at risk of increasing, owing to an under-appreciation of heterogeneity in local context^3–5^ and the ever-growing demands on the healthcare system, with fewer resources provided to manage them. As a result, quality and safety in healthcare, a discipline which aims to integrate scientific understanding with applied practice, has made significant progress over recent decades and is now regarded as an active and established community of researchers and practitioners alongside the fields of improvement and implementation science.^6–8^

Such growth has been reflected in the establishment of discipline-specific journals. For example, the BMJ launched *BMJ Quality and Safety* in 1992, and in 2006 a journal devoted purely to implementation science was introduced – *Implementation Science.* The evolution of the discipline has also included the development and refinement of a number of methodological tools, such as Plan, Do, Study, Act (PDSA) cycles and Driver Diagrams, which draw on the manufacturing industry to support individuals in applying continuous quality improvement (CQI) principles in healthcare practice.^9^

Despite the growing international interest in quality and safety in healthcare, its application to a mental health context has not been explored.^10^ It cannot be assumed that findings based on physical health in acute care hospitals can be automatically applied to mental health. This is because of the different challenges presented by patients and settings in this specialised area of care, including a greater emphasis on community-based care, greater use of Mental Health Act legislation and increased risk of self-harm.^10^ Mental health in general has been viewed as a neglected area and one in which patients may be less likely to have a voice when it comes to their care and safety.^11^ It has also been suggested that the stigma surrounding mental health issues has the potential in itself to contribute to staff neglecting patient safety and quality of care.^10^ In order to deliver high-quality care to patients, it is essential that a firmer understanding of patient safety and quality of care in mental health is not only developed, but also disseminated appropriately to ensure that it has the greatest impact.

Key literature searches of high-profile quality and safety journals reveal that there is a lack of published literature under the umbrella term of mental health. For example, a high-level search conducted in *BMJ Quality and Safety* in July 2016 based on the search term ‘mental health’ appearing in the title or abstract returns just 56 hits across all archives. When restricted to ‘mental health’ appearing in the title only (and therefore indicating that it is the primary focus of the article), the search returns just 17 results. This is disappointing, especially when compared with similar searches on key search terms for other medical specialties, for example paediatrics (94 hits for title and abstract) and surgery (237 hits for title and abstract). These findings are also reflected in other notable quality and safety journals such as *Implementation Science* (30 hits for ‘mental health’ in a title-only search) and the *International Journal for Quality in Health Care* (15 hits for ‘mental health’ in a title-only search). We recognise that there are inherent challenges in these comparisons, including selection of terminology and disciplines; however, these searches are intended to be illustrative rather than exhaustive.

Even the small number of studies that are returned from these searches do not consistently focus on mental health as the primary setting of interest. Instead, mental health tends to form one component of a system-level study often associated with high-level quality improvement and quality of care structures in the healthcare system.^12–14^ In other instances, mental health is positioned as just one example or context alongside physical health settings and is therefore not the sole focus of the article or its key messages.^15–17^ Generally, the work being published in these quality and safety journals does not focus on aspects of safe care that may be of specific importance to a mental health setting or explore how established quality and safety metrics apply and translate to this unique context. However, searches do identify a systematic review on medication errors in mental health^18^ and some work around continuity of care and communication between in-patient and out-patient mental health settings, for example.^19^

Contrary to these findings, searches run across the broader medical and social science literature reveal that much has been published on the topic of quality and safety in mental health in other, more specialty-specific areas (e.g. psychiatric nursing journals). For example, academic teams in mental health led by Louis Appleby, Len Bowers and Joy Duxbury contributed a significant amount of work. Therefore, it seems that the issue is not necessarily a lack of work on quality and safety within a mental health context, but instead a lack of its representation as part of the stand-alone quality and safety discipline.

The specialty-specific literature succeeds at providing a significant amount of research into patient safety incidents that are more precisely related to a mental health setting. These include violence and aggression, patient victimisation, suicide and self-harm, seclusion and restraint, and absconding and missing patients.^10^ Other key areas that apply more broadly across all settings are falls and other patient accidents, adverse medication events and adverse diagnostic events such as misdiagnosis. This literature is not without its faults, however, as there is a tendency for it to focus on areas of safety that may be of greatest concern to the public rather than areas of quality that may contribute most to patient experience and clinical outcome effectiveness. It may also not be fully reflective of the vast developments that have been made in understanding quality and safety in healthcare more broadly.

There is a clear disparity between the two bodies of literature (i.e. work around mental health within the established quality and safety discipline and work around quality and safety of care within the broader and less defined mental health discipline). In recent years there has been a call for ‘parity of esteem’ between physical and mental health (i.e. recognition of mental health as an equally important discipline within medicine).^20,21^ The data that we have presented certainly suggest that there is no parity in the attention being paid to quality and safety, and this is an area that requires attention. The structure of the National Health Service (NHS) is guilty of fostering this separation, to some extent, through commissioning different organisations to provide physical and mental healthcare.^22^ However, Academic Health Science Networks are aiming to help break down historical barriers between acute care and mental health trusts.

Furthermore, the two bodies of literature appear to exist in silos and do not explicitly refer to or build on one another as a matter of course. Therefore, the core integration of the quality and safety discipline with the mental health setting is currently lacking and not fully reflective of the scientific understanding that has been incrementally built up via the specialty-specific journals. The opportunity has also been missed for the two bodies of work to effectively communicate, learn from each other's limitations and strengthen one another. For example, a more thorough integration could ensure that quality and safety is explored across the board within the mental health setting in a way that is appropriately sensitive to the local context without being restrictive. This approach is likely to have the greatest direct benefit to mental health patients when such research translates into clinical practice.

It is important to discuss and reflect on the potential reasons for this disparity in order to understand how it might be rectified in the future. It is possible that academics focusing specifically on quality and safety as a research area (i.e. not wedded to any particular specialty) are not conducting a sufficient amount of research in a mental health context. Assuming that academics of this type are more likely to submit to quality and safety rather than specialty-specific journals, it is possible that the issue centres on a lack of work being completed in these settings by patient safety and quality improvement researchers.

A recent independent report into the quality of in-patient mental health services highlighted the need for further training and use of quality improvement in mental health services.^23^ The Royal College of Psychiatrists also recognise this issue and have set up a working group to steer progress. The Institute for Healthcare Improvement (IHI) is working with a number of mental health trusts in the UK to build capacity and capability to implement quality improvement programming at scale. It is important to recognise the challenges in applying improvement science in different healthcare delivery models, targeting different health conditions that follow very different courses. For example, the challenge of adapting quality improvement methodology for long-term conditions (which is often the case in a mental health setting) as opposed to interventional healthcare where it is simpler to measure impact and change pre- and post-implementation.

We must also consider what drives authors to publish in specialty-specific rather than quality and safety journals. It may be the case that mental health professionals and academics are more motivated to do so. For example, they may have concerns about ensuring that their work has the greatest impact or be unaware of the alternative journals that are appropriate. If this is the case, then raising awareness across the scientific community will be vital for ensuring that authors submit their work to the most suitable outlet in terms of target audience and opportunities for translation. On a separate note, it is possible that work is already being submitted to quality and safety journals but is not being accepted. There may be factors around quality of work and acceptance processes that need to be considered. This could be due to differences in academic approach and levels of rigour across the disciplines.

These dilemmas have a number of potential implications for both research and practice, and recommendations for the future are required in order to increase and support integration between the two bodies of work. Both the quality and safety and mental health disciplines should be concerned by the clear disparities between their bodies of work. Existing in silos automatically forms a barrier to effective quality improvement and safer patient care. Mental health should form a core part of the quality and safety agenda and influence the ways in which it grows and develops as a discipline over time. The disparity may also prevent the academic expansion of the discipline as a science owing to a lack of incremental growth that is fully reflective of all relevant research on this complex topic area. It is also likely that the mental health community will miss out on full access to the knowledge that has been accumulated within the quality and safety discipline, which will therefore prevent optimal patient care.

Quality and safety journals should explicitly invite submissions from the mental health community in order to demonstrate their openness to work based in this setting. Simultaneously, mental health professionals and academics should be made aware of the different disciplines with which they could be integrating their work, and should not be penalised for publishing their work in quality and safety rather than specialty-specific journals. The long-term goal should be to normalise that quality and safety journals are a viable option for mental health professionals' academic work. This would involve incorporating and building on the present understanding of quality and safety that has already been developed more broadly, rather than scoping out a separate area of quality and safety that applies solely to the mental health setting. Patient safety and quality of care in mental health should not be existing in a world of its own but instead be a fully integrated component of the broader scientific discipline. It is the responsibility of members of both communities to ensure that this happens.
